# D-Lactate Increases Cytokine Production in Bovine Fibroblast-Like Synoviocytes via MCT1 Uptake and the MAPK, PI3K/Akt, and NFκB Pathways

**DOI:** 10.3390/ani10112105

**Published:** 2020-11-13

**Authors:** Carolina Manosalva, John Quiroga, Stefanie Teuber, Sebastián Cárdenas, María Daniella Carretta, Gabriel Morán G, Pablo Alarcón, María Angélica Hidalgo, Rafael Agustín Burgos

**Affiliations:** 1 Faculty of Sciences, Institute of Pharmacy, Universidad Austral de Chile, Valdivia 5090000, Chile; carolinamanosalva@uach.cl; 2 Laboratory of Inflammation Pharmacology, Faculty of Veterinary Science, Institute of Pharmacology and Morphophysiology, Universidad Austral de Chile, Valdivia 5090000, Chile; john.quiroga@uach.cl (J.Q.); steubervolke@gmail.com (S.T.); sebastian.carvar@gmail.com (S.C.); daniellacarretta@gmail.com (M.D.C.); gmoran@uach.cl (G.M.G.); pabloalarcon.u@gmail.com (P.A.); 3 Laboratory of Immunometabolism, Faculty of Veterinary Sciences, Institute of Pharmacology and Morphophysiology, Universidad Austral de Chile, Valdivia 5090000, Chile; 4 PhD Program in Veterinary Sciences, Faculty of Veterinary Sciences, Universidad Austral de Chile, Valdivia 5090000, Chile

**Keywords:** ruminal acidosis, D-lactate, fibroblast-like synoviocytes, monocarboxylate transporter, Interleukin 6, Interleukin 8

## Abstract

**Simple Summary:**

Lameness is commonly observed in dairy cattle, affecting animal welfare and farm production. Ruminal acidosis is a metabolic disorder that frequently has been associated with lameness. Has been observed that during acute ruminal acidosis increase the presence of D-lactate in synovial fluid and induces the appearance of a joint inflammation, characterized by an aseptic synovitis. The fibroblast-like synoviocytes is one the main synovium cells involved in the onset of joint inflammation. The aim of this study was to assess the proinflammatory effect of D-lactate on bovine fibroblast-like synoviocytes. We demonstrated that D-lactate increase the release of proinflammatory cytokines, activating intracellular signaling pathways in bovine synoviocytes. Our results support the role of D-lactate in the onset of synovitis and lameness in cattle.

**Abstract:**

Acute ruminal acidosis (ARA) is caused by the excessive intake of highly fermentable carbohydrates, followed by the massive production of D-lactate and the appearance of neutrophilic aseptic polysynovitis. Bovines with ARA develop different lesions, such as ruminitis, polioencephalomalacia (calves), liver abscess and lameness. Lameness in cattle with ARA is closely associated with the presence of laminitis and polysynovitis. However, despite decades of research in bovine lameness as consequence of ruminal acidosis, the aetiology and pathogenesis remain unclear. Fibroblast-like synoviocytes (FLSs) are components of synovial tissue, and under pathological conditions, FLSs increase cytokine production, aggravating inflammatory responses. We hypothesized that D-lactate could induce cytokine production in bovine FLSs. Analysis by qRT-PCR and ELISA revealed that D-lactate, but not L-lactate, increased the expression of IL-6 and IL-8 in a monocarboxylate transporter-1-dependent manner. In addition, we observed that the inhibition of the p38, ERK1/2, PI3K/Akt, and NF-κB pathways reduced the production of IL-8 and IL-6. In conclusion, our results suggest that D-lactate induces an inflammatory response; this study contributes to the literature by revealing a potential key role of D-lactate in the polysynovitis of cattle with ARA.

## 1. Introduction

Acute ruminal acidosis (ARA) or D-lactic acidosis is one of the most severe forms of fermentative disorders in cattle and is caused by an excessive intake of highly fermentable carbohydrate-rich feed [1,2]. D-lactate is usually present in the blood of mammals in nanomolar concentrations due to methylglyoxal metabolism [3,4]. However, in ruminants, an overload of grains leads to the proliferation of carbohydrate-metabolizing bacteria, such as *Streptococcus bovis* and *Lactobacillus* spp. [5,6]. The main products of this metabolism are D and L-lactate, which lead to a consequent decrease in ruminal pH and an increase in lactate-producing bacteria [5]. D-lactate is the predominant enantiomer in the blood of cows with ARA, reaching concentrations of approximately 5 mM [7]. This concentration of D-lactate leads to a deep D-lactic acidemia, and D-lactate distribution to other anatomic compartments that has been associated with the appearance of clinical signs (e.g., diarrhea, depression with weakness, ataxia, coma, tarso-crural joints distention and lameness) [8,9,10,11]. Heifers subjected to experimental ARA by the administration of an oligofructose overload develop generalized sterile polysynovitis [1], which is a clinical disorder that is clearly underestimated in cattle lameness during ruminal acidosis [8,11]. The aseptic polysynovitis observed in ARA is characterized by the presence of abundant neutrophils and D-lactate concentrations of approximately 6 mM in the synovial fluid [8,9].

Fibroblast-like synoviocytes (FLSs) or type B synoviocytes are mesenchymal cells of the synovial tissue that possess many characteristics of fibroblasts [12]. These cells ensure the structural integrity of the synovial lining and secrete the components of the synovial fluid that are responsible for lubricating the joint [12]. However, under pathological conditions, FLSs produce mediators that induce angiogenesis, cell growth, leukocyte recruitment and immune cell activation, contributing to the exacerbation of the inflammatory response [13,14,15,16,17]. During aseptic joint inflammatory processes, FLSs produce high concentrations of lactate, which has been proposed to be crucial in the intracellular signaling pathway that controls the production of proinflammatory cytokines [18]. An increase in lactate, such as in the cases of acute abdomen disorders, hepatic and renal failure, and diabetic ketoacidosis, is considered a warning sign [19,20]. Recently, it has been shown that D-lactate increases neutrophil adhesion to endothelial cells by a mechanism that is dependent on the formation of neutrophil extracellular traps (NETs) [21]. Moreover, monocarboxylate transporter 1 and 2 (MCT1 and MCT2) inhibitors reduce the effects of D-lactate on neutrophils, suggesting that D-lactate needs to be transported into the cells to exert its proinflammatory effects.

In cattle with sterile synovitis induced by ARA, a massive presence of neutrophils and the release of aggregated neutrophil extracellular traps (aggNET) has been observed in synovial fluid [8]. IL-8 is the main cytokine chemoattractant of granulocytes that increased in lamellae tissue in cattle with ARA induced by oligofructose [22] and could be associated with granulocytes-recruitment observed in dermal lamellae [22,23].

Various inflammatory components, such as MMP-9, PGE2, IL-1β, and IL-6, have been found in the synovial fluid from cattle with ARA, being the latter the most abundant cytokine [8]. Similarly, in LPS-induced synovitis and lameness in horses, IL-6 is the higher proinflammatory cytokine found in synovial fluid [24]. The mitogen-activated protein kinase (MAPK) and nuclear factor-κB (NF-κB) pathways have been shown to play a predominant role in the expression of proinflammatory cytokines in joint inflammation [25,26]. In addition, bovine IL-6 [27,28] and IL-8 [29] genes contain promoter regions to NF-KB, being mainly upstream regulated by phosphatidylinositol 3-kinase (PI3K) pathway in synoviocytes [30,31].

Since the concentration of D-lactate in the synovial fluid is increased before the recruitment of neutrophils in cows with ARA [9], and IL-6 and IL-8 are the main cytokines that increase in those animals, we hypothesized that D-lactate promotes the expression of IL-6 and IL-8 and is dependent on the activation of MAPK, PI3K, and NF-κB in bFLS cells, thus contributing with the inflammatory process observed in synovial tissue [8,9].

## 2. Materials and Methods

### 2.1. Fibroblast-Like Synoviocyte Isolation and Culture

Fibroblast-like synoviocytes were isolated from the carpometacarpal joint of five Black Friesian multiparous dairy cows, 400–500 kg b.w., obtained from a local slaughterhouse. Joints without injuries such as synovial fluid turbid, presence of fibrin, mucopurulent secretions or hemorrhage, were used. The sample size used in all experiments was estimated from the results obtained previously in bFLS treated with D-lactate [9]. Briefly, we considered an effect size of 3.35, power = 0,95, and alpha = 0.05. A minimum sample size of 4 samples for group was calculated using G Power 3.1. Experiments were conducted in accordance with the recommendations of Agencia Nacional de Investigación y Desarrollo based on Chilean Animal Protection Laws. The study protocol was endorsed by the ethical committee of Universidad Austral de Chile (0023/18; Valdivia, Chile).

The synovial membrane was obtained and first digested with 0.2% type IV collagenase (Thermo Fisher Scientific, Waltham, MA, USA) for 2 h at 37 °C with gentle shaking. The suspension cells were seeded in a 75 cm^2^ flask for propagation. Dulbecco’s modified Eagle’s medium/Nutrient Mixture F12 (DMEM/F12; Thermo Fisher Scientific) containing 10% fetal bovine serum (FBS; Biowest, Nuaillé, France) was used. The cells were incubated with 5% CO_2_ at 37 °C. FLSs of passages 2–6 were used for the experiments. To confirm the isolation of bFLSs, CD14 (Becton Dickinson, Franklin Lakes, NJ, USA) and Vimentin (Acris, Herford, Germany) antibodies were used, and the expression of these markers was compared with that in a commercial bFLS line (Articular Engineering, Northbrook, IL, USA). Vimentin is a marker of FLSs and was expressed in both cell lines (Figure S1A) [32]. On the other hand, the absence of CD14 is an FLS marker. Bovine neutrophils (bPMNs) were used as a positive control (Figure S1B) [33]. The isolation of bPMNs was performed as described by Mena et al. [34].

### 2.2. Quantitative Real-Time PCR

bFLSs were seeded in 6-well plates and were treated with vehicle (0.1% DMSO or H2O), 2 mM or 5 mM D-lactate, 2 mM or 5 mM L-lactate (Sigma-Aldrich, St. Louis, MO, USA) or 100 ng/mL bovine TNF-α (bTNF-α) (Thermo Fisher Scientific) for 6 h at 37 °C and 5% CO2. Prior to stimulation, the cells were incubated with vehicle (0.1% DMSO) or different inhibitors, namely, 10 µM LY294002 (Sigma-Aldrich) [35,36], 10 µM UO126 [36], 10 µM BAY 11-7082 (Cayman Chemicals, Ann Arbor, MI, USA) [37], 10 µM SB203580 [38], or 1 µM Ar-c 155858 (Tocris, Bristol, UK) [21], for 30 min. The supernatant was collected and stored for cytokine detection. Total RNA was extracted from the bFLSs using RNA Kit I (Omega Bio-Tek Inc., Norcross, GA, USA) according to the manufacturer’s protocol. RNA was treated with Turbo DNAse (Ambion, Thermo Fisher Scientific) to ensure the removal of genomic DNA. Three hundred nanograms of RNA was reverse transcribed using M-MLV Reverse Transcriptase (Promega, Madison, WI, USA). Real-time PCR was performed in a StepOnePlus™ (Applied Biosystems, Life Technologies, Foster City, CA, USA) using the Takyon™ Rox SYBR^®^ master mix (Eurogentec, Seraing, Belgium), and the primers are listed in Table 1. The following conditions were used: 40 cycles at 95 °C for 30 s, 60 °C for 30 s (annealing), and 72 °C for 30 s (extension); for IL-6, the annealing temperature used was 55 °C. The expression levels of the cytokines and MCTs were normalized to the expression of the housekeeping gene RPS-9 and then quantified using the 2^−ΔΔCT^ method, according to Livak and Smittgen [39], using StepOne™ v2.3 software (Applied Biosystems).

### 2.3. Immunoblot

bFLSs were seeded in 6-well plates and were stimulated with 5 mM D-lactate for 0, 2, 5, 10, 15 min or 100 ng/mL bTNF-α for 5 min at 37 °C and 5% CO_2_. Total proteins were obtained as previously described [34], and 30 μg of protein was separated using 12% SDS-PAGE gels and transferred onto nitrocellulose membranes. The membranes were blocked with 5% skim milk in TBS/T (20 mM Tris–HCl, pH 7.6, 137 mM NaCl, and 0.05% Tween 20) and incubated with anti-MCT1 and anti-MCT4 antibodies (Bioss Antibodies Inc., Woburn, MA, USA) or with phospho-ERK1/2, phospho-p38 or phospho-Akt antibodies (Cell Signaling, Beverly, MA, USA) overnight at 4 °C. Each membrane was incubated with an HRP-conjugated secondary antibody (LI-COR, Lincoln, NE, USA). The bands were visualized using an Odyssey Fc infrared/chemiluminescent detection system (LI-COR Biosciences). After signal detection, the membranes were stripped according to Hidalgo et al. [35] and reprobed with antibodies against total p38 MAPK, ERK1/2, Akt (Cell Signaling, Beverly, MA, USA) or β-actin-HRP (Santa Cruz Biotechnology, Santa Cruz, CA, USA). The band intensity was measured using Image Studio Lite v5.2 software (LI-COR Biosciences).

### 2.4. Quantification of Intracellular D-Lactate in bFLSs by HPLC

bFLSs were seeded in 6-well plates and were incubated with 5 mM D-lactate for 5 min at 37 °C and 5% CO_2_. After stimulation, the cells were lysed with liquid nitrogen and 500 μL of mobile phase (1 mM CuSO_4_). The lysate was centrifuged at 18,000× *g* for 20 min at 4 °C. The supernatant was collected, and the pellet was used for the quantification of total proteins. The supernatant was loaded into Amicon^®^ Ultra-4 3K (Merck Millipore, Darmstadt, Germany) tubes and centrifuged at 75,000× *g* for 40 min at 4 °C. The filtrates were concentrated in a SpeedVac concentrator (Savant^®^ SPD131DDA, Thermo Fisher Scientific) for 90 min at 45 °C and 1.5 atmospheres of pressure. The concentrates were suspended in 200 µL of 1 mM CuSO_4_ and analyzed using HPLC, as previously described [21].

### 2.5. Determination of IL-6 and IL-8 by ELISA

The supernatants obtained from the stimulated bFLSs for the qPCR assay were used to estimate the concentration of proinflammatory cytokines by using bovine IL-8 (#3114-1A-6, Mabtech, Nacka, Sweden) and IL-6 ELISA kits (#ESS0029, Thermo Fisher Scientific) according to the manufacturer’s instructions. Briefly, the capture antibody was incubated overnight, and the wells were then blocked for 1 h; subsequently, 100 μL of sample was added and incubated for 1–2 h. After the plates had been washed twice, the detection antibody was added and incubated for 1 h. After two additional washes, streptavidin was added, and the mixture was incubated for an additional 0.5–1 h. Finally, p-nitrophenyl phosphate (pNPP) or tetramethylbenzidine substrate solution (TMB) was added, followed by incubation for 20–25 min in the dark. All the procedures were performed at room temperature. The reaction was stopped with 0.16 M H_2_SO_4_ (for IL-6 ELISA Kit), and the samples were analyzed at 405 and 450 nm for IL-8 and IL-6, respectively, in an automatic Varioskan Flash Reader (Thermo Fisher Scientific).

### 2.6. Determination of IκBα Levels by Flow Cytometry

bFLSs were seeded in 6-well plates and were stimulated with 5 mM D-lactate for 0, 15, 30, 45 min or 100 ng/mL bTNF-α for 15 min at 37 °C and 5% CO_2_. The cells were collected by centrifugation after trypsinization and were fixed with 4% paraformaldehyde for 15 min. Next, the cells were washed with PBS and permeabilized with 90% methanol ice-cold. To analyze the effect on the IκBα levels, the cells were incubated with an AlexaFluor 488-conjugated IκBα mouse mAb (Cell Signaling, Beverly, MA, USA) for 1 h. The cells were analyzed using a FACS Canto II flow cytometer (Becton Dickinson, Franklin Lakes, NJ, USA) and FlowJo 7.6 software (TreeStar Inc., Ashland, OR, USA).

### 2.7. Statistical Analysis

All the assays are shown as the mean ± SEM of 5 independent experiments. One-way analysis of variance (ANOVA) was performed, and Fisher’s LSD multiple comparison test was applied, using a significance level of 5%. When assumptions of normality or homogeneity of variance were not met according to the Shapiro–Wilks or Brown–Forsythe test, respectively, Kruskal–Wallis ANOVA and Dunn’s multiple comparison test were used. All the statistical analyses were performed using GraphPad Prism v7.0 (GraphPad Software, La Jolla, CA, USA). A *p*-value < 0.05 was considered significant.

## 3. Results

### 3.1. D-Lactate Increases the Expression of IL-6 and IL-8 in bFLSs

**Figure 1:**
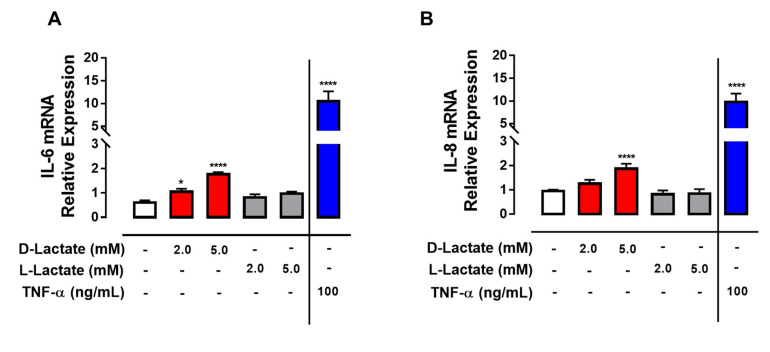
D-lactate increases the expression of IL-6 and IL-8 in bovine fibroblast like synoviocytes (bFLSs). Relative expression of IL-6 (**A**) and IL-8 (**B**) mRNA in bFLS cells treated with D-lactate or L-lactate for 6 h. Bovine tumoral necrosis factor alpha (bTNF-α) was used as a positive control. Each bar represents the mean ± standard error of media (SEM). *n* = 5, * *p* < 0.05; **** *p* < 0.0001 compared to control.

Previously, we demonstrated an increase in the lactate enantiomers in the joints of cows with ARA [8,9]. We observed that D-lactate induced a statistically significant increase in IL-6 and IL-8 mRNA (Figure 1A,B). However, the expression of CXCL-1, CXCL-2, CXCL-3, and CXCL-6 remained unchanged (Figure S2). On the other hand, when we evaluated the effect of L-lactate, there was no increase in the expression of any of the cytokines analyzed (Figure 1A,B; Figure S2). Additionally, the effect of Na+ D-lactate was evaluated, and we observed that Na+ D-lactate significantly increased the expression of IL-6 and IL-8 (Figure S3). bTNF-α was used as a positive control for the expression of proinflammatory cytokines in bFLSs, and we demonstrated an increase in the expression of IL-6, IL-8, CXCL-1, and CXCL-2 (Figure 1A; Figure S2). 

### 3.2. D-Lactate Uptake is Mediated by MCT1

**Figure 2:**
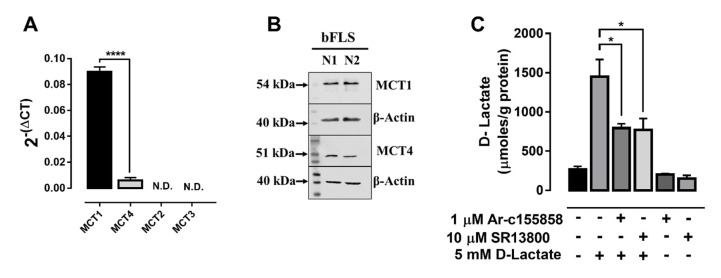
The uptake of D-lactate is mediated by monocarboxylate transporter 1 (MCT1). (**A**) The relative mRNA levels of MCT1, MCT2, MCT3 and MCT4 in bFLSs were normalized by S9 ribonucleoprotein (RPS9, housekeeping gene). (**B**) To confirm the presence of MCTs, immunoblotting of MCT1 and MCT4 in total bFLS proteins was performed. β-actin was used as a load control. (**C**) Intracellular levels of D-lactate in bFLSs stimulated for 5 min were measured by HPLC. In addition, two MCT1 inhibitors were used to confirm the transport of D-lactate, Ar-c155858 and SR13800, *n* = 5, * *p* < 0.05; **** *p* < 0.0001.

We observed an increase in the expression of IL-8 and IL-6 with D-lactate in bFLSs. Therefore, we hypothesized that bFLSs can express monocarboxylate transporters (MCTs) that mediate its bidirectional cotransport coupled to protons (H^+^) [40]. We observed that the mRNA of MCT1 and MCT4 were expressed, but MCT2 and MCT3 were not detected, in bFLSs (Figure 2A). Additionally, the relative abundances of MCT1 and MCT4 were different, and MCT1 was significantly more abundant than MCT4 (15-fold) (Figure 2A). In addition, the presence of MCT1 and MCT4 in bFLSs was corroborated by western blot (Figure 2B). Next, we evaluated the functionality of MCT1 in bFLSs. The influx of D-lactate in bFLSs was measured by assaying the intracellular contents of the enantiomers by HPLC. The stimulation of bFLSs with 5 mM D-lactate for 5 min increased the intracellular concentration of D-lactate in comparison with cells treated with the vehicle alone (water) (Figure 2C). Additionally, the uptake of D-lactate was reduced by pretreatment of bFLSs with Ar-c155858 [41], which is a potent inhibitor of MCT1 and MCT2, and SR13800, which is a selective inhibitor of MCT1(Figure 2C) [42]. 

### 3.3. Inhibition of MCT1 Reduced the Expression of IL-6 and IL-8 Induced by D-Lactate

**Figure 3:**
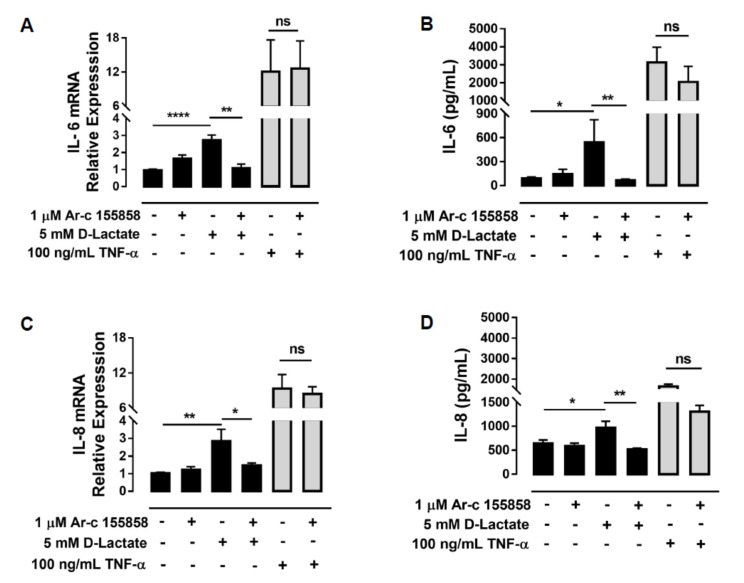
The influx of D-lactate by MCT1 increases the expression of IL-6 and IL-8. (**A**,**C**) The relative mRNA levels of IL-6 and IL-8 in bFLSs pretreated with Ar-c 155858 and then stimulated with D-lactate or bTNF-α were normalized by S9 ribonucleoprotein (housekeeping gene). (**B**,**D**) The IL-6 and IL-8 concentrations in the supernatants of bFLSs treated with Ar-c 155858 and stimulated with D-lactate or bTNF-α were measured by ELISA. The mean ± SEM of five independent experiments is shown. NS: not significant. * *p* < 0.05, ** *p* < 0.01, **** *p* < 0.0001 compared with control or D-lactate-treated cells.

The role of MCT1 in IL-8 and IL-6 expression induced by D-lactate was assessed. bFLSs were incubated with 1 µM Ar-c155858 (inhibitor of MCT1 and MCT2) and then stimulated with 5 mM D-lactate. Cotreatment with this inhibitor resulted in a significant reduction in the mRNA levels of IL-6 and IL-8 in comparison to D-lactate treatment alone (Figure 3A,C). Additionally, we observed a decrease in the secretion of both cytokines by ELISA (Figure 3B,D). To confirm the specificity of this inhibition, we evaluated the effect of Ar-c155858 on the expression of IL-6 and IL-8 induced by bTNF-α. The treatment of bFLSs with Ar-c155858 did not change the expression and synthesis of both cytokines induced by bTNF-α (Figure 3A–D). 

### 3.4. ERK1/2, p38 MAPK and PI3K/Akt Signaling Pathways Mediate IL-6 and IL-8 Production Induced by D-Lactate

**Figure 4:**
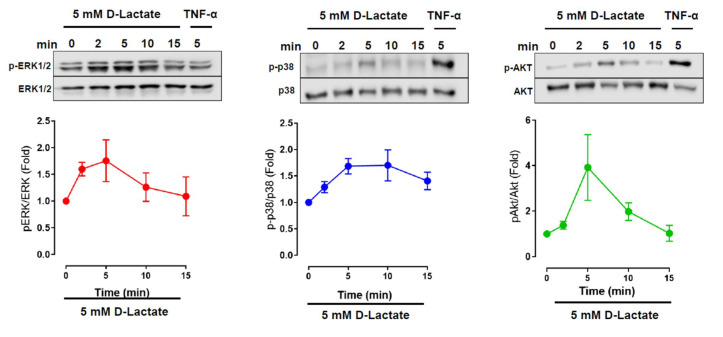
D-lactate increases the phosphorylation of ERK1/2, p38 and Akt in bFLSs. bFLSs were treated with 5 mM D-lactate at a range of time points (0, 2, 5, 10 and 15 min). Total protein was analyzed by sodium dodecyl sulfate polyacrylamide gel electrophoresis (SDS/PAGE) and immunoblotting using specific antibodies against the phosphorylated forms of ERK1/2, p38 and Akt. Total ERK1/2, p38 and Akt were also evaluated by western blot for comparison. The images of one representative experiment are shown. The densitometry ratios of phospho extracellular signal-regulated kinase/extracellular-regulated kinase (pERK/ERK), p-p38/p38 and p-Akt/Akt are shown in the graphs (*n* = 3).

**Figure 5:**
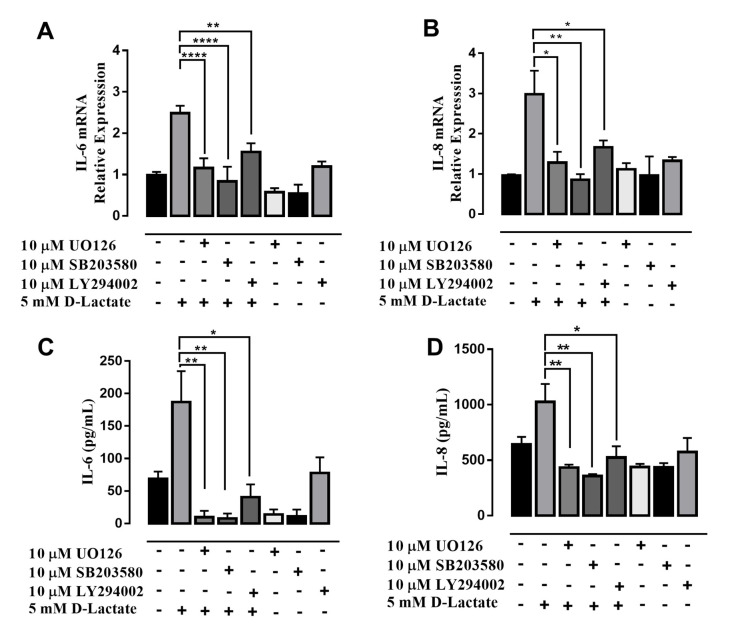
ERK1/2, p38 and Akt participate in the increase in IL-6 and IL-8 induced by D-lactate in bFLSs. (**A**,**B**) The relative mRNA levels of IL-6 and IL-8 in cells treated with UO126, SB203580 and LY294002 and then stimulated with D-lactate, normalized by S9 ribonucleoprotein (housekeeping gene), are shown. (**C**,**D**) The IL-6 and IL-8 concentrations in the supernatants of bFLSs treated with UO126, SB203580 and LY294002 and stimulated with D-lactate were measured by ELISA. The mean ± SEM of five independent experiments is shown. * *p* < 0.05, ** *p* < 0.01, **** *p* < 0.0001, compared with control or D-lactate-treated cells.

To investigate the cytoplasmic signaling involved in the regulation of IL-6 and IL-8 mediated by D-lactate in bFLSs, we evaluated the phosphorylation of ERK1/2, p38 and Akt. Compared with the control, we observed that stimulation of bFLSs with 5 mM D-lactate resulted in increases in the phosphorylation of these proteins, which peaked at 5 min of stimulation in all cases (Figure 4 and Supplementary Figure S4). In addition, bTNF-α was used as a control for phosphorylation. To determine whether these kinases are involved in the increase in IL-6 and IL-8 expression induced by D-lactate, the pharmacologic inhibitors UO126, SB203580 or LY294002 were used. According to the results, the D-lactate-induced elevation in the IL-6 and IL-8 mRNA and protein levels were decreased by UO126, an inhibitor of MEK1/2 kinases whose function is to phosphorylate ERK1/2 (Figure 5A–D). Additionally, the inhibitor of p38 (SB203580) and the PI3K/Akt inhibitor (LY294002) reduced the expression and secretion of IL-6 and IL-8 (Figure 5A–D). Overall, our results suggest that D-lactate increases the expression and secretion of IL-6 and IL-8 in a manner that depends on the activation of the ERK1/2, p38 and Akt pathways.

### 3.5. The Expression of IL-6 and IL-8 Induced by D-Lactate Is Dependent on NF-κB Activation

**Figure 6:**
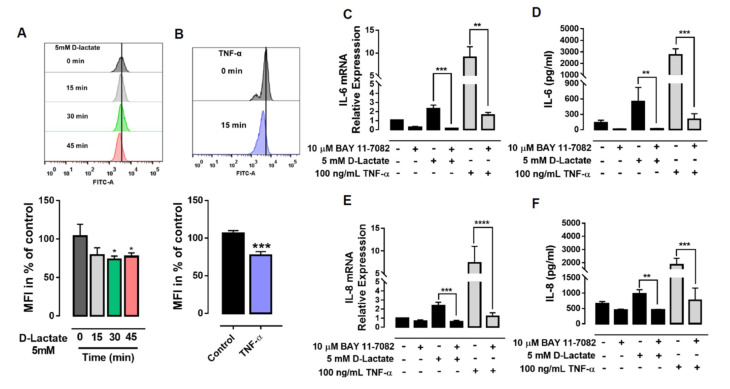
NF-κB regulates the increase in the expression and secretion of IL-6 and IL-8 induced by D-lactate in bFLSs. IκBα degradation was evaluated in cells treated with (**A**) D-lactate and (**B**) bTNF-α by flow cytometry. The cells were treated with BAY 11-7082, an NF-κB inhibitor, and then treated with D-lactate or bTNF-α. (**C**,**E**) The mRNA levels of IL-6 and IL-8 normalized by RPS9 in cells treated with D-lactate or bTNF-α are shown. (**D**,**F**) The concentrations of IL-6 and IL-8 in cells treated with D-lactate or bTNF-α are shown. The mean ± standard error of media (SEM) of five independent experiments is shown. * *p* < 0.05, ** *p* < 0.01; *** *p* < 0.001; **** *p* < 0.0001 compared with control or D-lactate-treated cells.

We observed that D-lactate significantly induced the degradation of IκBα after 30 min of stimulation (Figure 6A). bTNF-α was used as a positive control, and according to our experiments, we observed that bTNF-α induced the degradation of IκBα at 15 min (Figure 6B). Next, we evaluated the effect of BAY 11-7082, which irreversibly inhibits IKKα and thus reduced the phosphorylation and degradation of IκBα, resulting in the inactivation of NF-κB [43]. We observed a significant decrease in the expression and secretion of both cytokines induced by D-lactate (Figure 6C–F). Similarly, we observed that the expression and secretion of these cytokines induced by bTNF-α diminished significantly when the cells were pretreated with BAY 11-7082 (Figure 6C–F). 

## 4. Discussion

Bovines with ARA develop different clinical signs, such as ruminitis, polioencephalomalacia (calves), liver abscess and lameness [5,44,45]. In particular, lameness in cattle with ARA is closely associated with the presence of laminitis and aseptic neutrophilic polysynovitis [5,11,46,47,48]. However, despite decades of research on bovine lameness as a consequence of ruminal acidosis, the etiology and pathogenesis remain elusive [2,47,49,50]. During ARA, an increase in lactate in the ruminal fluid and plasma is observed [51]. Moreover, Morrow et al. (1973) were successful in experimentally inducing laminitis in lambs by intraruminal injection of lactic acid [52], suggesting that lactate could be involved in cattle lameness. Recently, several metabolic changes in the synovial fluid of cows with ARA and polysynovitis have been described, including an increases in L-lactate, D-lactate, and fructose and decreases in citric acid and threonine; moreover, the presence of D-lactate in the joint could contribute to the onset of synovitis in cattle [8,9]. In support of this, a proinflammatory effect of D-lactate has been described in neutrophils [21,53]; however, the effects of D-lactate on synovial tissue have not yet been described.

We demonstrated that D-lactate increases the expression and release of IL-6 and IL-8 in bFLSs. IL-6 activates the immune system, favoring the inflammatory response, and in the joint, IL-6 expression correlates positively with the intensity of the lesions observed in aseptic joint diseases, such as rheumatoid arthritis (RA) [54,55,56]. In accordance with this, we previously demonstrated that D-lactate and IL-6 exhibit early increases in the synovial fluid of heifers with ARA and are increased before joint neutrophil recruitment [8]. Thus, IL-6 is considered a key player in joint inflammatory diseases [56,57]. In addition, FLSs may also interact with endothelial cells to promote neutrophil recruitment, which is inhibited by antibodies against IL-6, suggesting that the IL-6 produced by FLSs may increase the expression of adhesion molecules and chemokines by endothelial cells, facilitating neutrophil adherence [58].

IL-8 is a chemoattractant cytokine essential in neutrophil-mediated inflammation [59,60,61,62,63,64]. IL-8 increases the expression of CD11b in bovine neutrophils, promoting their migration to inflamed tissues [65,66]. In the synovial fluid of RA patients, IL-8 promotes leukocyte infiltration, and these leukocytes, once activated, release a variety of proteinases and enzymes, such as collagenase, elastase, myeloperoxidase and MMP-9, thus contributing to joint damage [67,68]. Previously, we observed an increase in the infiltration of neutrophils and MMP-9 in the synovial fluid of cows with ARA [8]; however, in the synovial fluid of animals with ARA, the presence of IL-8 has not yet been detected. We demonstrate that bTNF-α, a well-known joint proinflammatory agent [12,69,70,71], increases the production of IL-6 and IL-8 in bFLSs in a similar fashion to that described in hFLSs [72]. bTNF-α, but not D-lactate, increased the expression of CXCL-1 and CXCL-2. In this context, our results suggest that D-lactate could play a key role in the polysynovitis observed during ARA through the synthesis of IL-6 and IL-8 by bFLS. 

Both stereoisomers, L-lactate and D-lactate, can exert several proinflammatory effects [18,73], including NET formation [21,73]. In synovial fluid induced by ARA, the presence of NET has been reported [8]. D-lactate induces NET formation and dependent of its uptake via MCT-1, in bovine neutrophils. [21]. In addition, IL-8 and IL-6 have been described as NET inducers [74,75] and therefore, we did not discard that the release of these cytokines induced by D-lactate contributes to NET release observed during synovitis in cattle with ARA [8]. L-lactate increases the production of IL-8 in human endothelial cells [76] and IL-6 in human skeletal muscle cells [77]. Moreover, it has been proposed that the IL-6 and IL-8 production induced by TNF-α in RA-FLSs is dependent on L-lactate [18]. Surprisingly, we observed that L-lactate did not induce an increase in the production of proinflammatory cytokines in bFLSs. Other authors showed that D-lactate more strongly regulates the differentiation of mouse adipocytes compared to L-lactate [78], suggesting that D- and L-lactate can exert different effects based on the cellular type used. Furthermore, our results exclude a possible proinflammatory effect that has been attributed to pH, since the pKa of both enantiomers is 3.86 [3,79].

At physiological pH, both enantiomers are dissociated with a ratio of 3000:1 for lactate ions and lactic acid [3]. Lactate is transported into and out of cells via proton-dependent monocarboxylate transporters (MCTs) [3,80]. Some isoforms of MCTs have been described in bovine rumen epithelium [81] and bovine neutrophils [21]. In this research, we identified a higher expression of MCT1 compared with MCT4 in bFLSs. In contrast, RA-FLSs exhibit higher levels of MCT4 than MCT1, and similar expression levels are observed in osteoarthritis FLSs [82]. We demonstrated that D-lactate can be transported by bFLSs via MCT1. In addition, MCT1 inhibition blocked the expression and secretion of IL-6 and IL-8 induced by D-lactate. Hence, the current study provides the first evidence that MCT1 is involved in D-lactate entry in bFLSs, which is required for proinflammatory cytokine expression. In accordance with the above, it has been shown that endothelial cells can uptake lactate by MCT1, allowing the production of IL-8 [76]. Other authors demonstrated that the uptake of lactate through the MCT1 transporter increased COX-2 expression in monocytes [83] and regulated the migration of CD 8^+^ T cells [53]. Moreover, inhibition of D-lactate influx reduced the NET release and endothelial adhesion induced by D-lactate in bovines [21].

We observed that D-lactate increased the phosphorylation of ERK1/2 and p38, in bFLSs, being these proteins pertaining to the MAPK family. Other authors have suggested that lactate could activate ERK1/2 and p38 MAPK in RA-FLSs [18]. Therefore, our results are the first to show that D-lactate activates the MAPK signaling pathway in bFLSs. In addition, the inhibition of these kinases decreased IL-6 and IL-8 expression. p38 MAPK and ERK1/2 regulate the synthesis of IL-6, IL-12, IL-23 and TNF-α in RA-FLSs [84,85,86]. MAPKs play important roles in mediating synovial inflammation and joint destruction, and they are considered critical molecular targets for therapeutic intervention in joint inflammation [18,87].

Our results also suggest that D-lactate and bTNF-α activate the PI3K/Akt pathway in bFLSs. Moreover, the inhibition of PI3K/Akt reduced the expression and secretion of IL-6 and IL-8 induced by D-lactate. Similarly, other authors have described that TNF-α can induce the expression of IL-6 and IL-8 via PI3K/Akt in human FLSs [30,70].

The ERK1/2, p38 and PI3K/Akt pathways regulate the nuclear translocation of NF-κB, which is essential in proinflammatory cytokines production in human FLSs [88,89,90,91,92]. In connection with this, NF-κB binding sites in the promoter region of the IL-6 and IL-8 genes in RA synovial tissue have been previously reported [93]. Thus, we tested the ability of D-lactate to induce cytokine expression via the NF-κB pathway. Our experiments demonstrated that D-lactate and bTNF-α increase the degradation of IκBα in bFLSs. Moreover, the pharmacological inhibition of NF-κB reduced the expression of IL-8 and IL-6 induced by D-lactate and bTNF-α. Other authors have shown that lactate increases the degradation of IκBα, promoting the translocation of NF-κB to the nucleus in human FLSs and endothelial cells [18,76]. In human FLSs, TNF-α induces the phosphorylation and degradation of IκBα [94], permitting NF-κB translocation into the nucleus to activate the transcription of target genes [43]. Additionally, Georganas et al. demonstrated that the activation of NF-κB is key for the constitutive secretion of IL-6 and IL-8 as well as the secretion of these cytokines induced by IL-1β in RA-FLSs [95]. Altogether, our results strongly suggest that D-lactate increases the release of pro-inflammatory cytokines, activating intracellular signaling pathways in bovine synoviocytes, supporting the role of D-lactate in the onset of synovitis in cattle with ARA. 

A dairy herd with ruminal acidosis showed higher ruminal D-lactic acid production associated with an increase in swollen fetlocks and laminitis [96]. Similarly, other authors observed arthritis in cattle that was defined as “laminitis with concurrent general affection”, in addition, the total contents of protein and leukocytes was increased in the synovial fluid from fetlock joints [97]. Furthermore, ARA induced experimentally in cattle produces aseptic synovitis and lameness [1,8]. Our results contribute to propose D-lactate as a metabolic pro-inflammatory agent for the joint of cattle. However, in vivo experiments are required to demonstrate the role of D-lactate in bovine lameness.

## 5. Conclusions

The present study revealed that in bFLSs the influx of D-lactate, through MCT1, induces the ERK1/2, p38, Akt, and NF-κB signaling pathways that promote the expression and secretion of IL-6 and IL-8. 

## Figures and Tables

**Table 1:** Sequences of forward and reverse primers.

Gene	Forward Primer Sequence (5′-3′)	Reverse Primer Sequence (5′-3′)	Size (Bp)
bCXCL-1	CCGCCCCCATGGTTAAGAAA	AAACACAGTCCAGATGGCCC	161
bCXCL-2	CCAGCTCTAACTGACCAGGTG	ATGGCCTTAGGAGGTGGTGA	116
bCXCL-3	GCCATTGCCTGCAAACTT	TGCTGCCCTTGTTTAGCA	189
bCXCL-6	ATTCATCCCAAAACGGTCAGTG	CAGACTTCCCTTCCATTCTTCAAG	101
bIL-6	ACTGGCAGAAAATAAGCTGAATCTTC	TGATCAAGCAAATCGCCTGAT	89
bIL-8	ATGACTTCCAAGCTGGCTGTTG	TTGATAAATTTGGGGTGGAAAG	149
bMCT-1	CGCCGCGAGCCGCGTATAA	CCTCCAACTGCTGGTGGCATTGT	85
bMCT-2	CCACCCAGTGCCGGAGACCA	TCCCGTGTCTAAGGTTGCCCAGG	70
bMCT-3	GAGGCTGTGGCTGTGCTCATCG	GATCTCGTAGTTCTTGAGCGCGTCC	72
bMCT-4	ATCCAGCAAGCCCTCCCTTCCC	CCATGGCCAGGAGGGCTGATTCT	100
bRPS9	GCTGACGCTGGATGAGAAAGACCC	ATCCAGCACCCCGATACGGACG	85
bRPS9	TTCCAGAGCGTTGGCTTAGG	ACCCTCCAGACCTCACGTTT	181

## Supplementary Materials

The following are available online at https://www.mdpi.com/2076-2615/10/11/2105/s1, Figure S1. Expression of Vimentin and CD14 in bFLSs and neutrophils, respectively. (A) To confirm the isolation of bFLS, the marker vimentin was used, and the expression of vimentin was compared with that of a commercial line of bFLS. (B) The absence of CD14 is an FLS marker, and bovine neutrophils (bPMNs) were used as a positive control. Figure S2. Expression of proinflammatory cytokines in bFLS. Relative expression of CXCL-1, CXCL-2, CXCL-3 and CXCL-6 mRNA in bFLSs treated with D-lactate, L-lactate or bTNF-α for 6 h. ** *p* < 0.01; *** *p* < 0.001 compared with the control. Figure S3. Na^+^ D-lactate increases the expression of IL-6 and IL-8 in bFLSs. Relative expression of IL-6 and IL-8 mRNA in bFLS cells treated with Na^+^ D-lactate for 6 h. Each bar represents the average ± S.E.M. *n* = 5. * *p* < 0.05 compared to the control. Figure S4. D-lactate increases the phosphorylation of ERK1/2, p38 and Akt in bFLSs. bFLSs were treated with 5 mM D-lactate during 5 min. Total protein was analyzed by SDS/PAGE and immunoblotting using specific antibodies against the phosphorylated forms of ERK1/2, p38 and Akt. Total ERK1/2, p38 and Akt were also evaluated by western blot for comparison. TNF-α (100 ng/mL) was used as a positive control. The images of one representative experiment are shown.
