# Supplementary material for: D-Lactate Increases Cytokine Production in Bovine Fibroblast-Like Synoviocytes via MCT1 Uptake and the MAPK, PI3K/Akt, and NFκB Pathways
Source: Animals (Basel). 2020 Nov 13;10(11):2105. doi: 10.3390/ani10112105 (PMC7698040; doi:10.3390/ani10112105)

Supplementary Fig. 1

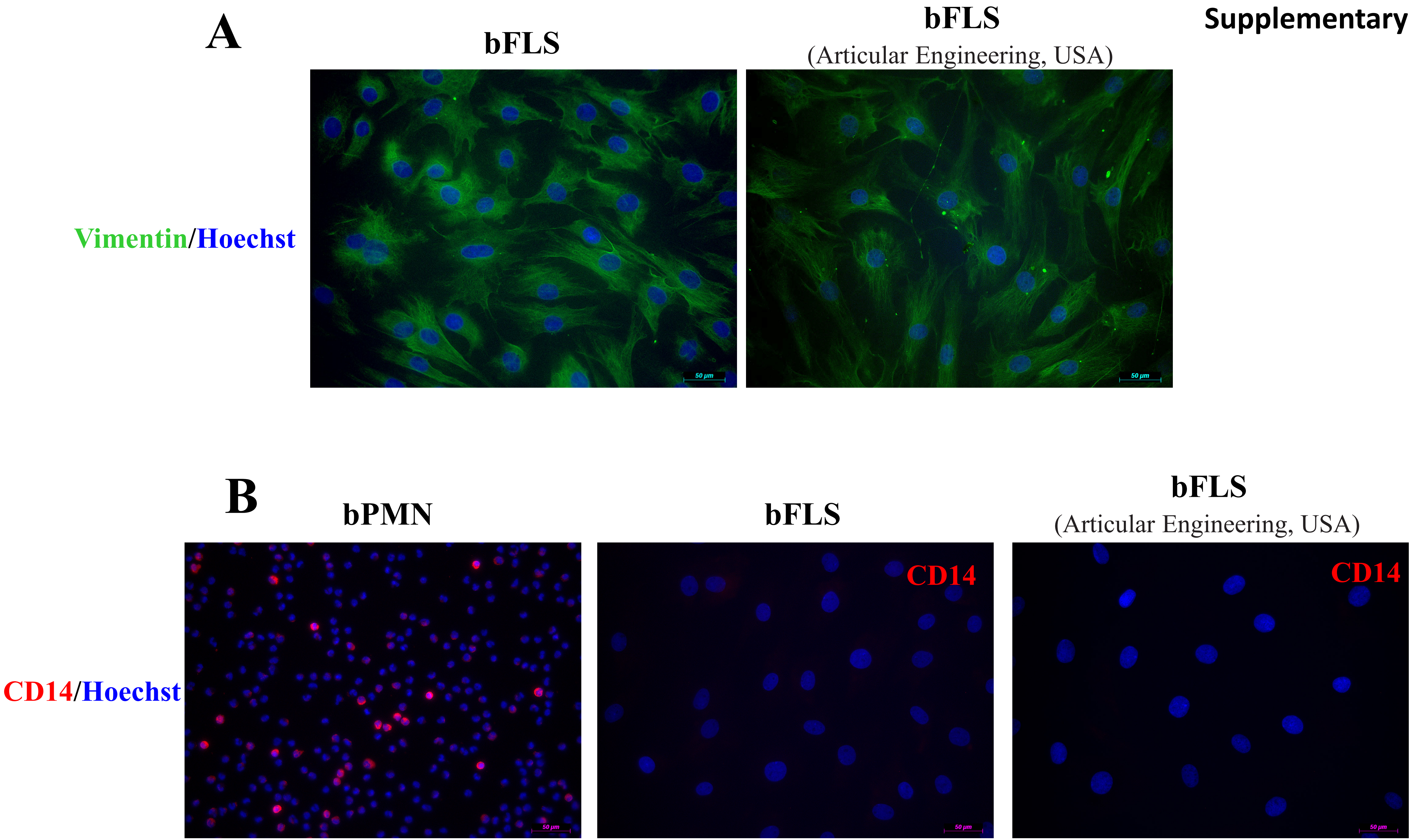

Supplementary Fig. 2

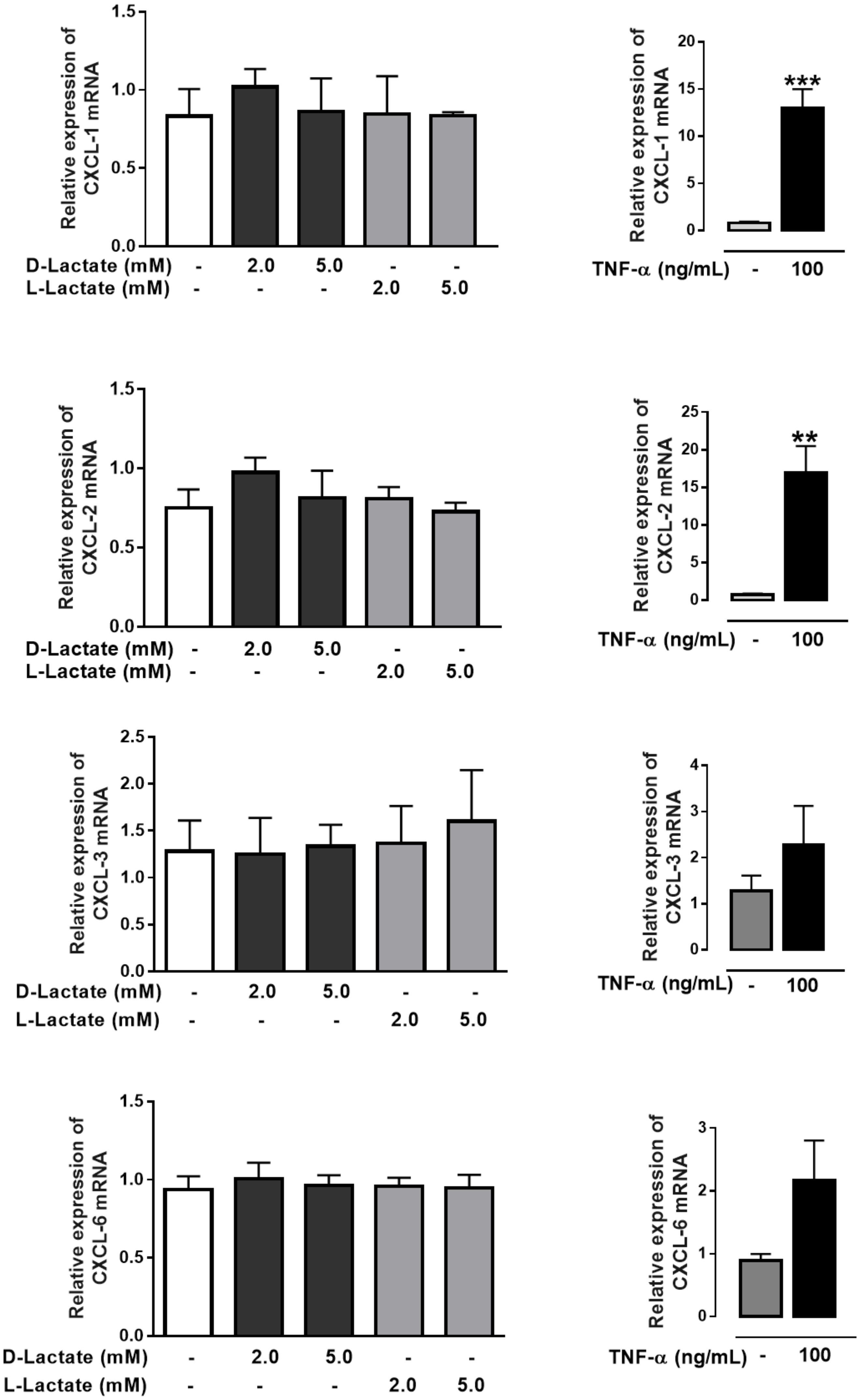

Supplementary Fig. 3

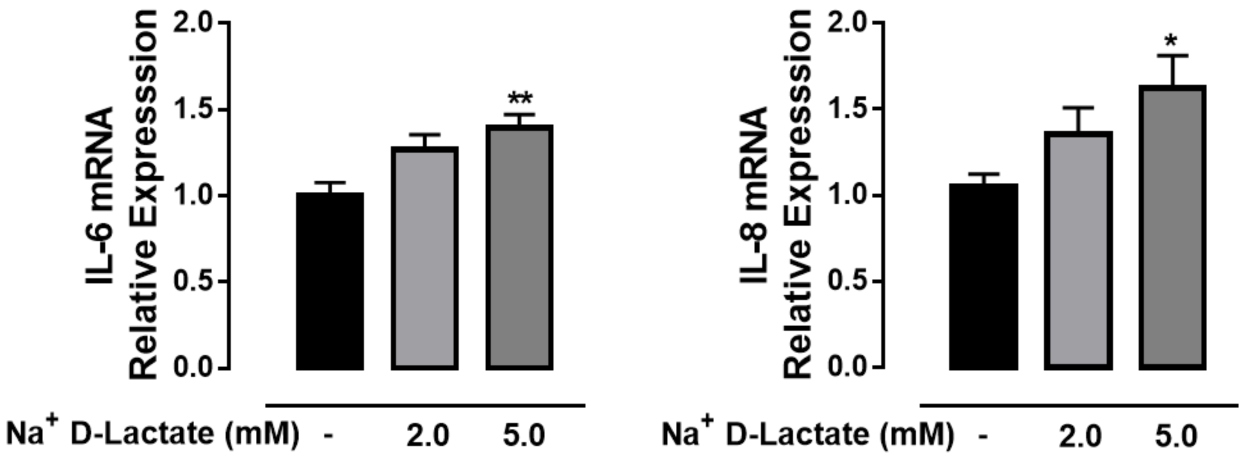

Supplementary Fig. 4

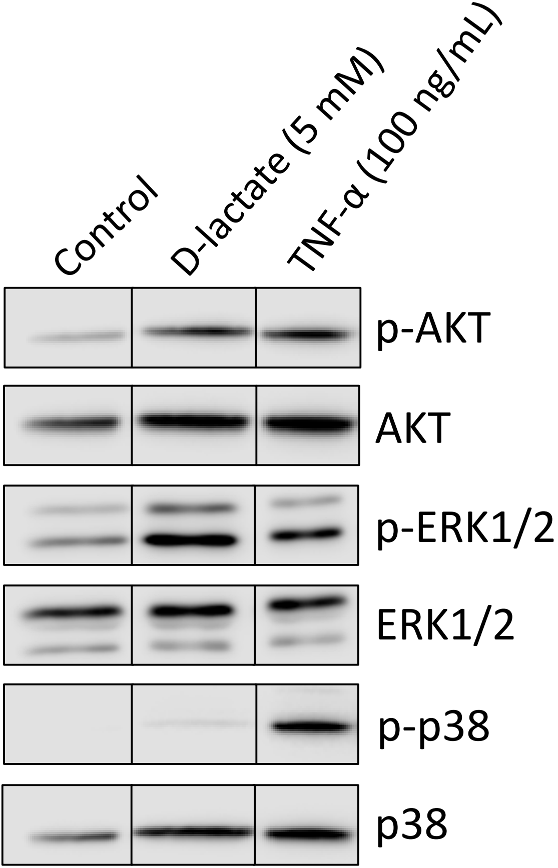

Supplement: Supplementary file 1 [file animals-10-02105-s001.pdf]
